# Low-Light Image Enhancement Based on Generative Adversarial Network

**DOI:** 10.3389/fgene.2021.799777

**Published:** 2021-11-29

**Authors:** Nandhini Abirami R., Durai Raj Vincent P. M.

**Affiliations:** School of Information Technology and Engineering, Vellore Institute of Technology, Vellore, India

**Keywords:** computer vision, deep learning, facial expression recognition, convolutional neural network, human-robot interaction, generative adversarial network

## Abstract

Image enhancement is considered to be one of the complex tasks in image processing. When the images are captured under dim light, the quality of the images degrades due to low visibility degenerating the vision-based algorithms’ performance that is built for very good quality images with better visibility. After the emergence of a deep neural network number of methods has been put forward to improve images captured under low light. But, the results shown by existing low-light enhancement methods are not satisfactory because of the lack of effective network structures. A low-light image enhancement technique (LIMET) with a fine-tuned conditional generative adversarial network is presented in this paper. The proposed approach employs two discriminators to acquire a semantic meaning that imposes the obtained results to be realistic and natural. Finally, the proposed approach is evaluated with benchmark datasets. The experimental results highlight that the presented approach attains state-of-the-performance when compared to existing methods. The models’ performance is assessed using Visual Information Fidelitysse, which assesses the generated image’s quality over the degraded input. VIF obtained for different datasets using the proposed approach are 0.709123 for LIME dataset, 0.849982 for DICM dataset, 0.619342 for MEF dataset.

## Introduction

Cameras are crucial in capturing real-world happenings in several accomplishments like remote sensing, autonomous driving solutions, and surveillance systems. Computer vision algorithms used for these applications require the images to be high visibility to achieve commendable performance (Lore et al., 2017). However, the quality of the images captured is greatly affected by the amount of light received by the camera’s sensor. Also, the low-light images are prone to have additional noise (Wang et al., 2020). Hence, high-quality images cannot be obtained under low-light conditions that affect the performance of computer vision applications like object detection, recognition, segmentation and classification (Ai and Kwon, 2020). Thus, developing a low-light image enhancement technique is essential to perform subsequent high-level computer vision tasks with ease and high accuracy. Recently, deep learning-based approaches gained popularity in many computer vision applications (Abiram et al., 2021; Abirami et al., 2021) as they achieved good results. The performance of conditional generative adversarial networks in computer vision-based applications (NandhiniAbirami et al., 2021) inspired us to explore the performance in low-light image enhancement. The experimental performance on the synthetic and real-world low-light image datasets demonstrates the robustness of the method.

**FIGURE 1:**
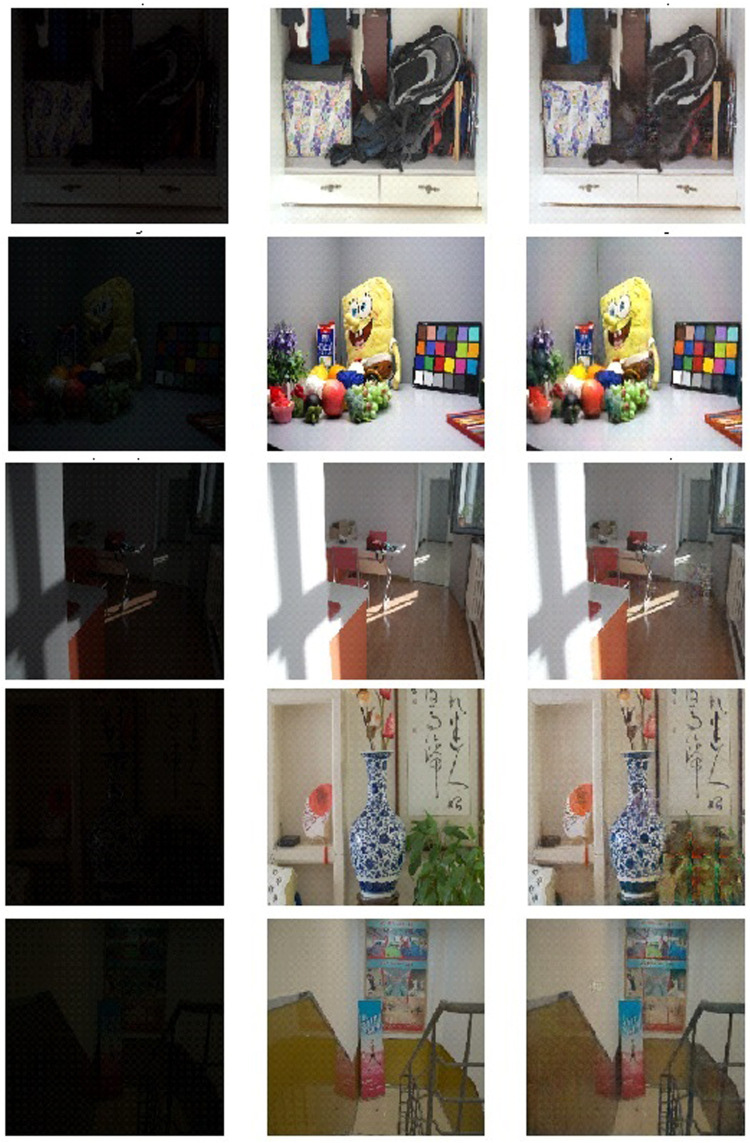
**Left Column:** Natural low-light images. **Middle Row:** Ground truth images. **Right Column:** The results enhanced by our method.

Figure 1 showcases several examples of the image enhancement by the proposed method. It can be seen wall paintings fourth and fifth image, shadow and reflection on the floor in the third image are accurately captured and generated by the model. The proposed model brought out to light the intricate details that have almost been buried in the dark. As shown in Figure 1, our method works best visually, and the results are very similar to ground truth. To bring the buried information to light, image enhancement is necessary (Lv et al., 2021). Intensifying the image directly is the simplest and the inherent approach to bringing the low-light regions into the light. But, amplifying the low light regions paves the way to other problems like enhancing the naturally bright regions to get saturated and lose intricate details (Ke et al., 2020; Xie et al., 2020).

Several other existing methods for low-light image enhancements are performed using image dehazing (Li et al., 2015) Retinex theory (Park et al., 2017; XiaojieGuo et al., 2016; Park et al., 2018; Shi et al., 2019; Zhang et al., 2018) and histogram equalization algorithm (Singh et al., 2015; Xu et al., 2013; Celik, 2014; Arya and Pattanaik, 2013). The histogram equalization technique avoids saturation and it is also one of the widely used techniques for its effectiveness and simplicity. However, the histogram equalization technique enhances the low-light image utilizing the histogram details of the entire low-light image as its transformation function, resulting in excessive enhancement of the regions with a low level of contrast. In other words, the technique focuses on enhancing the contrast rather than performing actual illumination, causing under or over-enhanced regions in the image. The approach presented in (Wang et al., 2013) enhances contrast and retains the illumination in the image but fails to retain the image’s visual quality.

According to retinex theory, the amount of light reaching the sensor is composed of illumination and scene reflection represented by,

E = R∘T (1)

Where, E is the image irradiance, which is the illumination reaching the camera sensor, R is the reflection map and T is the illumination map while ∘ representing elementwise multiplication. Some methods obtained enhanced results by removing the illumination part (Wang et al., 2014), while some methods retained a part of the illumination to maintain the natural effects of the images (Wang et al., 2013). However, these methods introduce distortions to the enhanced images leading to reduced visual quality by ignoring the camera response characteristics. Methods based on image dehazing retain pixel values to maintain natural distribution. As observed in (Dong et al., 2011) proposed an approach to perform image dehazing on the input low visibility image. After this, image inversion is performed on the unnatural images to obtain the illuminated image. Although the above methods produced good results, those images did not reflect the true illumination and contrast of the scene. Some of the methods do not take into account the effect of noise in the resulting images. Also, some methods will get different results for different illuminations. Therefore, we propose a conditional generative adversarial network-based model to enhance and denoise the degraded low-light image in this work (Mirza and Osindero, 2014). This work

1) Rigorously analyses the efficiency of GAN in image-based tasks.
2) Presents a low-light image enhancement technique (LIMET) with a fine-tuned conditional generative adversarial network preserving the naturalness of the image.
3) Presents an approach that employs two discriminators to acquire a semantic meaning that imposes the obtained results to be realistic and natural.

## Related Works

Generally, the image enhancement technique improves the visual quality of the low-quality low-light images. It supports further high-level computer vision tasks to extract valuable information from the captured images (Jung et al., 2017). The low-light image enhancement technique unveils the poorly exposed regions of the image. Many kinds of research have been proposed to improve the visibility of images regardless of the lighting conditions.

Based on the retinex theory, several models were proposed for low-light image enhancement (Fu et al., 2018). Diverse methods were proposed to separate the illumination component from the given image. SSR(Si et al., 2017), MSR, and MSR with color restoration (Ma et al., 2017) techniques have been popularly employed as image enhancement techniques. Guo et al. (XiaojieGuo et al., 2016) calculated the illumination of all the pixels by choosing the greatest value among RGB channels. The work non-uniformly enhanced the illumination instead of eliminating the color shift posed by light sources. Though these methods successfully extract the illumination invariant features in the estimated reflectance, the over-highlighted edges and color inconsistency often make the enhanced images visually unnatural and significantly degrades the viewing experience. To overcome these issues, methods have been proposed to apply the dehazing algorithm. Dong et al. (Lv et al., 2021) proposed a dehazing based image enhancement algorithm. The method shows the illumination element of the image the inverted low-light version of the image. The enhanced image is acquired by inverting the unrealistic estimated image once again (Dong et al., 2011). Fu et al. (2016a) proposed a fusion-based method where an illumination estimation algorithm to decompose the image into illumination and reflectance components. Then, contrast-enhanced and illumination-improved versions are derived from the illumination component by utilizing adaptive histogram equalization and sigmoid function. Although the fusion-based method provided promising results, it struggles to produce textual details across multiple scales.

The main focus of the histogram equalization algorithms is enhancing image contrast and balancing the histogram of the whole image as much as possible. It is frequently used as a prerequisite for object recognition and detection (Sellahewa and Jassim, 2010). However, the details hidden in the dark region are not enhanced properly as this method considers each image pixel individually, disregarding their neighborhood. This makes the image inconsistent with the real scene. Celik et al. (Celik and Tjahjadi, 2011) tried to overcome these issues by proposing variational methods that use various regularization terms on the histogram. The authors enhanced the contrast using the variational methods that mapped the elements between the histogram of the actual and the smoothened image. However, several dehazing methods can provide only reasonable results. Deep learning based Unsupervised GAN proposed by Yifan et al. (Jiang et al., 2021) that trains the model without image pairs captured under low-light and normal light. The authors used the information obtained from the input instead of using the ground truth image. Ning et al. (Rao et al., 2021) proposed a component GAN model to recover normal images from poor light images. The proposed network is composed of two components namely decomposition part and enhancement part. The decomposition part divides the low light images into illumination and reflectance component. The enhancement part generates good quality images.

Several techniques have been put forward for low-light image enhancement, but it still suffers from the over or under-enhanced images due to poor image visibility. Recently studies have been proposed to preserve the illumination structure by minimizing the color effects. Still, these methods suffer from loss of textural details resulting in smoothed out surfaces of objects. In contrast, the proposed model has a clear physical explanation preserving textural details of the objects in the image. Technical aspects of the LIMET model will be discussed below. Table 1 shows the advantages and disadvantages of low-light image enhancement techniques.

**TABLE 1:** Advantages and disadvantages of existing low-light image enhancement techniques.

References	Method	Dataset	Advantages	Disadvantages
Petro et al. (2014)	Multiscale retinex	Dataset collected from multiple sources	Images are enhanced under varied illumination conditions. Color restoration restores wrong colors	The method introduces artifacts and noises in the image resulting in a blurry image
Xiaojie Guo et al. (2016)	LIME: Low light image enhancement *via* illumination map estimation	High-dynamic range dataset	The algorithm is computationally inexpensive	The bright regions are over-enhanced and lose contrast
Li and Wu (2017)	Learning-based restoration of backlit images	Data obtained from Li’s database	Backlit regions are identified and enhance the degraded image	Processing takes a long time, and the enhanced image quality depends highly on the accuracy of segmentation
McCann and Vonikakis (2018)	Estimating the retinal contrast from the image	High-dynamic range dataset	It illuminates both low-light and brighter areas of the image	Underexposed regions are slightly enhanced

## Materials and Methods

**FIGURE 2:**
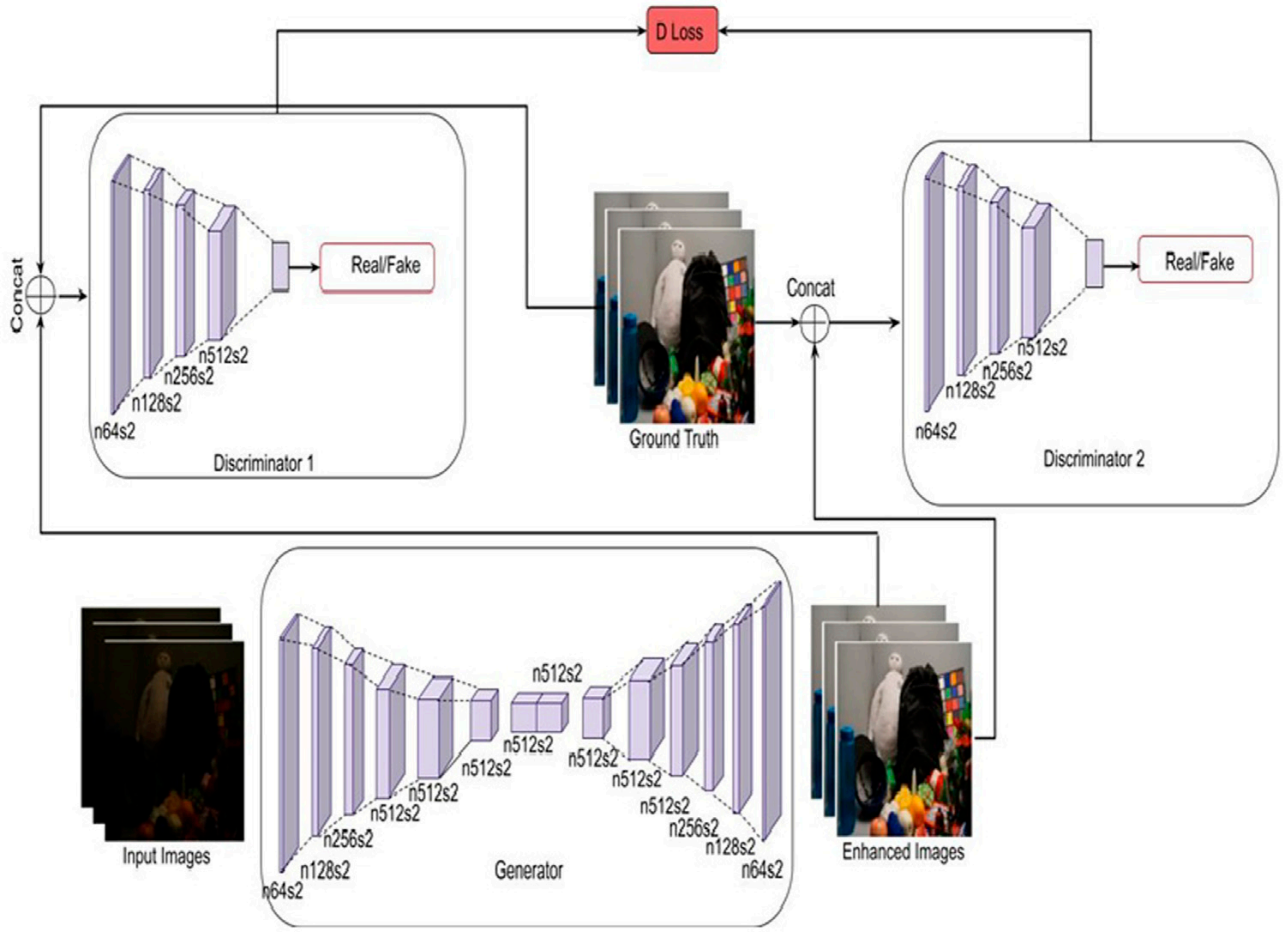
Proposed methodology.

The proposed approach, network structure, loss function, and implementation details are discussed in this section. Figure 2 presents the architecture of the proposed low-light image enhancement model. The model is proposed with a dual discriminator sharing a similar architecture but with varying sizes. The weights of the inputs passed to the two discriminators are the actual and 1/2 of the actual weight. This ensures that the discriminator guides the generator to generate realistic images and enhance the visual appeal of the input. This is because the existing discriminator does not guide generators to generate realistic or very close to realistic images. The discriminator model uses convolution-instance normalization-leaky ReLU blocks of layers. The convolutional layers have filters ranging from 64 to 512. Here, instance normalization is used instead of batch normalization to remove artifacts. The network architecture uses the Sigmoid activation function to determine the probability. The sigmoid activation function is most frequently used in classification problems. These dual discriminators are used to assist the generator in generating realistic images. The network is trained using an ADAM optimizer having a learning rate set to 0.0002, $\beta_{1}$ 0.5 and $\beta_{2}$ 0.5. The learning rate is fixed throughout the training. The batch size is set to 16.

The proposed methodology overcomes the disadvantages of classical image enhancement techniques, namely 1) retinex based methods that generate graying out and unnatural images, 2) unsharp masking algorithm that fails to achieve a good tradeoff between details and naturalness, 3) histogram equalization that fails to generate realistic images, 4) adaptive histogram equalization that does not produce effective results.

The generator not only reduces the loss from the discriminator but also generates the fake distribution close to the real distribution by using loss $L_{1}$ . In the optimization process, the generator loss is calculated using $L_{1}$ and sigmoid cross-entropy loss. $\;L_{1}$ loss is the difference between the ground truth and the generated image. $L_{1}$ loss is calculated to make the generator generate the images structurally similar to the ground truth. The final generated loss is the total of the mean absolute error $L_{m}$ and the sigmoid cross-entropy loss $L_{CE}\;$ represented by,

$$L_{CE}=\;-t_{i}\log s_{i}-\left(1-t_{i}\right)\text{log}\left(1-s_{i}\right)$$ (2)

$$L_{m}=\sum_{i=1}^{n}y_{true}\;-y_{predicted}$$ (3)

$$L_{G}=\;L_{m}+L_{CE}$$ (4)

$y_{true}$ is the Ground truth.

$y_{predicted}\;$ is the Generated image.

$s_{i}$ is the binary indicator denoting the class for the sample i.

$t_{i}$ is the predicted probability ranging between 0 and one for that sample i.

**FIGURE 3:**
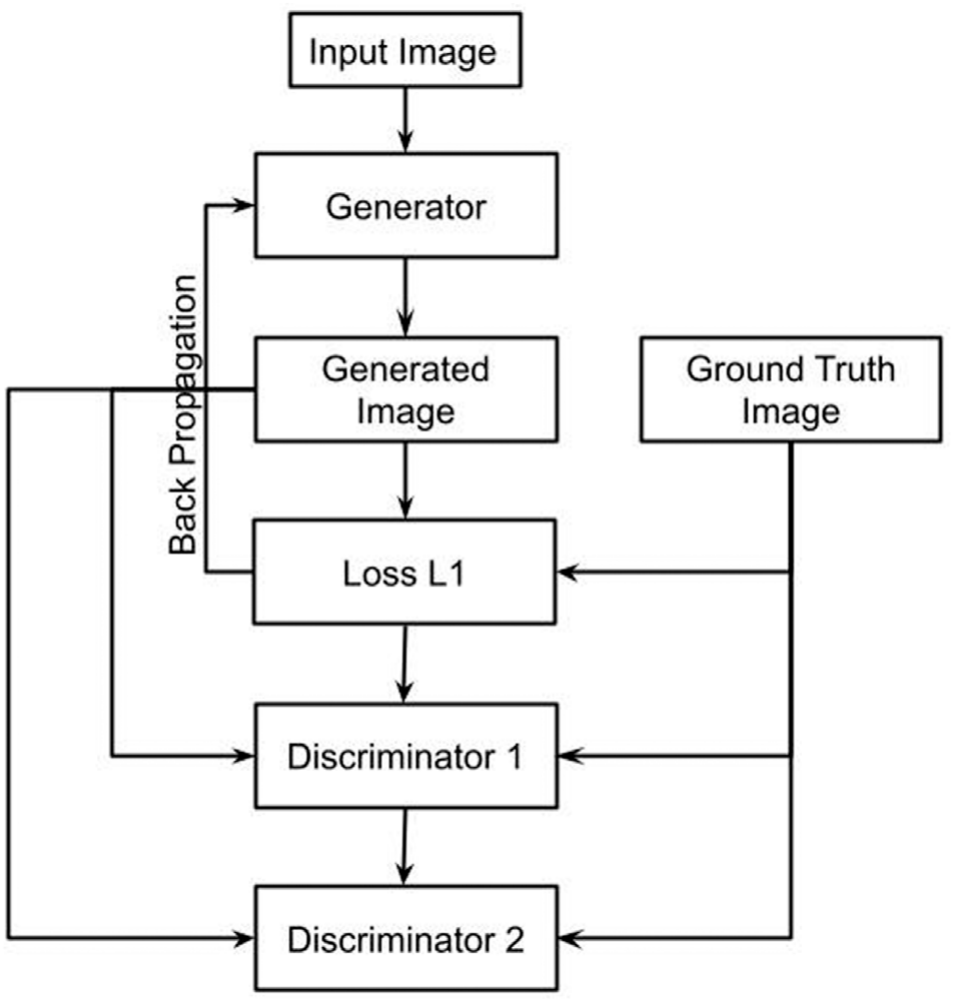
Flowchart of the proposed model.

Each discriminator block contains a convolution layer, instance normalization layer, and LeakyReLU represented by Eq. 5. The dual discriminator loss is calculated by taking in the actual and the fake images as the inputs. The discriminator loss is a summation of actual loss and generated loss. The real loss is a sigmoid cross-entropy loss of real images. The generated loss is a binary cross-entropy loss of fake images. Instance normalization is used to remove artifacts. A sigmoid activation function represented by Eq. 6 is used as a discriminator acts as a binary classifier classifying the real and fake images. A Sigmoid activation function converts any value to a 0–1 probability for two-class classifications. Figure 3 shows the flowchart for the proposed approach.

$$Sigmoid\;\sigma \left(x\right)=\;\frac{1}{1+e^{-x}}$$ (5)

Leaky ReLU = max(0.1x,x) (6)

Algorithm 1

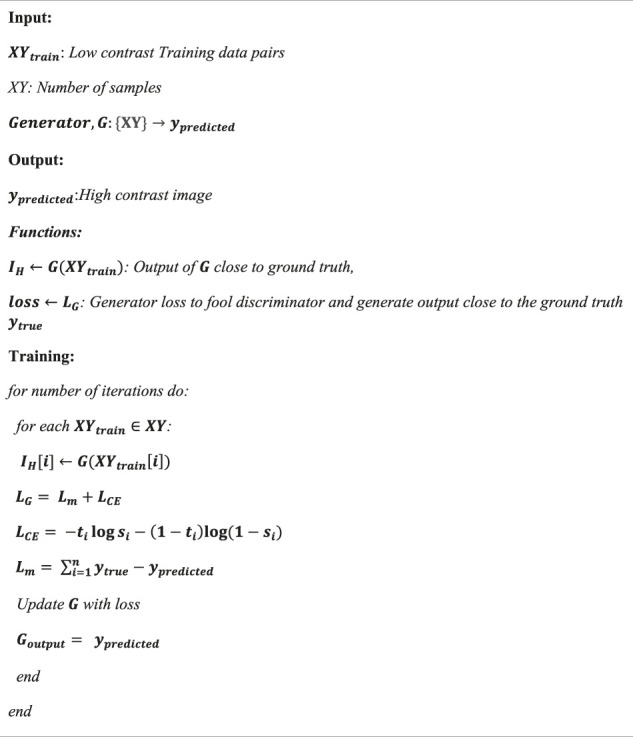

## Experimental Results

**FIGURE 4:**
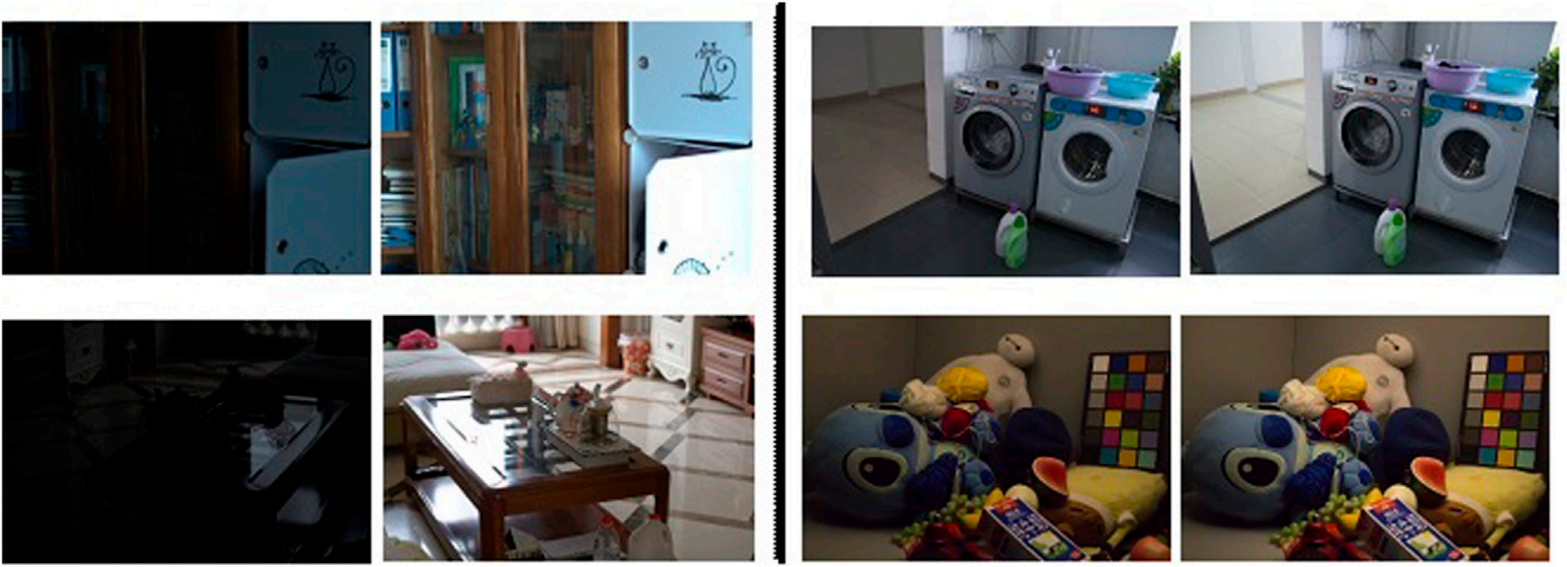
Samples of training data pairs showing low light image samples **(left)** and normal light image samples **(right)**.

The proposed model is trained using the LOL dataset (Wei et al., 2018), having 500 low-light image pairs. The image pairs in the LOL dataset are synthesized on real scenes. The ground truth images of the LOL dataset are normal light images. Figure 4 shows the degraded and the ground truth samples from the training data under different illumination conditions. In the training phase, a batch of paired low-light images is fed into the generator to learn the features of the image.

The results generated from LIMET for low-light image enhancement are compared with several exisiting state-of-the-art methods. The performance of the model is compared with Dehazing based fast efficient algorithm (Dong) (Dong et al., 2011), Naturalness preserved enhancement algorithm (NPE) (Wang et al., 2013), Low-light image enhancement *via* illumination map estimation (LIME) (Abiram et al., 2021), A fusion-based enhancing method (MF) (Fu et al., 2016a), Simultaneous Reflection and Illumination Estimation (SRIE) (Fu et al., 2016b), MultiscaleRetinex (Petro et al., 2014), GLobal illumination Aware and Detail-preserving Network (GLADNet) (Wang et al., 2018). The performance evaluations of these methods are performed using publicly available datasets, namely LIME (Abiram et al., 2021) dataset with ten low-light images, DICM(Lee et al., 2012) dataset 69 images captured by digital cameras, MEF (Ma et al., 2015) with 17 high-quality multi-exposure images that include natural scenaries and architectural pictures.

**FIGURE 5:**
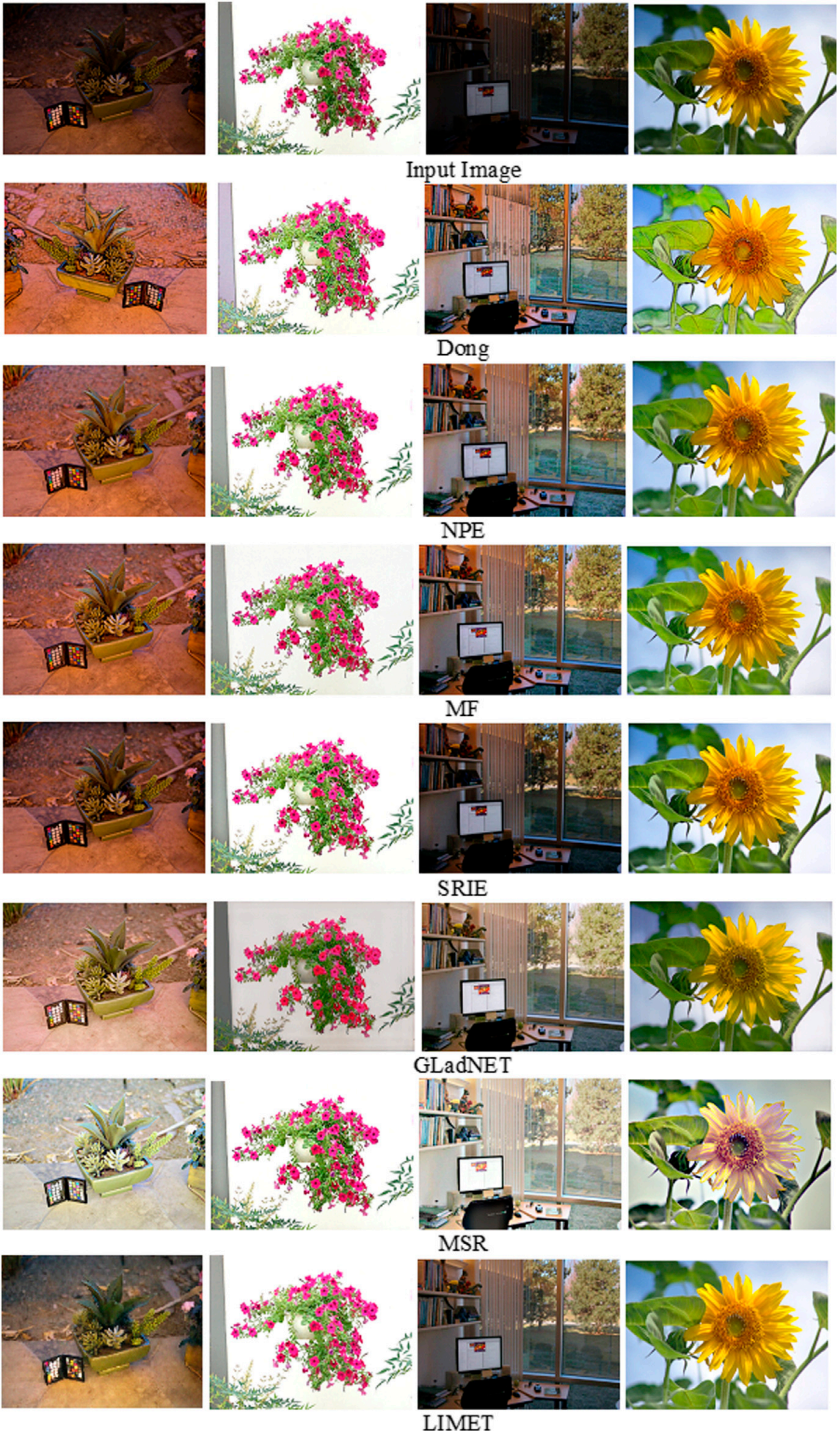
Enhancement comparison of the proposed approach with existing approaches.

It can be visualized from Figure 5 that the proposed approach generates high-quality results compared to the existing studies. The evaluation of the proposed approach with previous studies in terms of VIF is presented below. The experimental results reveal that the proposed model is more robust, effective and provides enhanced results on degraded images with less noise.

The performance of LIMET is compared with existing state-of-art-methods using Visual Information Fidelity. VIF is a measure of distortion of visual information used to evaluate the quality of the improved images. Fidelity is the similarity between the signals from the reference and distorted images. VIF is originally designed for degraded images, here as the images are enhanced, the original image is considered the degraded image. Hence, VIF is applied in reverse where the enhanced image, C is referred as the ground truth image and the original image as the degraded image, F. VIF is given by,

$$VIF=\;\frac{I\;\left(C;F\right)}{I\left(C;E\right)}$$ (7)

Here, E is the perceived image, I (C;F) and I(C;E) represent the data obtained from the ground truth image, C and distorted image, F.

From Table 2 it is visible that LIMET performs superior compared to the existing best models. The results depict that the existing methods do not adequately enhance image quality when compared to the proposed approach. The proposed approach achieved better outputs with improved visual quality and low distortion. The proposed approach generates robust results and performs better than the existing methods in terms of VIF.

**TABLE 2:** Summarizes the VIF results in the images compared to the state-of-the-art models.

Method	LIME	DICM	MEF
	Data	Data	Data
Dong[23]	0.309192	0.446071	0.288288
NPE[21]	0.528744	0.803247	0.434567
LIME[4]	0.492474	0.595954	0.407859
MF[28]	0.545735	0.732275	0.438101
SRIE[35]	0.655896	0.805968	0.602530
MSR[31]	0.483033	0.484776	0.409118
GLadNET[36]	0.447379	0.6369877	0.433680
LIMET	0.709123	0.849982	0.619342

## Conclusion

This work has presented a robust approach to improve the visual quality and offer vision-based applications with dependable inputs. The model is robust and the results obtained from LIMET can be fed to several computer vision-based applications like feature matching, edge detection, object recognition, with enhanced inputs improving their performances. Experimental results reveal that the proposed approach LIMET achieves visually enhanced and natural results that surpass the existing image enhancement methods. The proposed approach has a clear physical explanation preserving textural details of the objects in the image in comparison with other existing approaches. However, the proposed approach does not handle images with severe noise effects and in the future studies can be proposed to handle images with more noise. Future studies can utilize enhanced images for several applications, namely object detection, segmentation and classification.

## Data Availability

Publicly available datasets were analyzed in this study. This data can be found here: https://drive.google.com/file/d/157bjO1_cFuSd0HWDUuAmcHRJDVyWpOxB/view.

## Abbreviations

CGAN, conditional generative adversarial network; GLadNET, low-light enhancement network with global awareness; HE, histogram equalization; LIME, low-light image enhancement; LIMET, low-light image enhancement technique; LOL, low-light dataset; MSR, multiscaleretinex; NPE, naturalness preserved enhancement; ReLU, rectified linear unit; SSR, single scale retinex; SRIE, Simultaneous Reflection and Illumination Estimation; VIF, visual information fidelity.
